# Imatinib Ameliorates Neuroinflammation in a Rat Model of Multiple Sclerosis by Enhancing Blood-Brain Barrier Integrity and by Modulating the Peripheral Immune Response

**DOI:** 10.1371/journal.pone.0056586

**Published:** 2013-02-20

**Authors:** Milena Z. Adzemovic, Manuel Zeitelhofer, Ulf Eriksson, Tomas Olsson, Ingrid Nilsson

**Affiliations:** 1 Neuroimmunology Unit, Department of Clinical Neuroscience, Center for Molecular Medicine, Karolinska Institutet, Stockholm, Sweden; 2 Division of Vascular Biology, Department of Medical Biochemistry and Biophysics, Karolinska Institutet, Stockholm, Sweden; Innsbruck Medical University, Austria

## Abstract

Central nervous system (CNS) disorders such as ischemic stroke, multiple sclerosis (MS) or Alzheimeŕs disease are characterized by the loss of blood-brain barrier (BBB) integrity. Here we demonstrate that the small tyrosine kinase inhibitor imatinib enhances BBB integrity in experimental autoimmune encephalomyelitis, an animal model of multiple sclerosis (MS). Treatment was accompanied by decreased CNS inflammation and demyelination and especially reduced T-cell recruitment. This was supported by downregulation of the chemokine receptor (CCR) 2 in CNS and lymph nodes, and by modulation of the peripheral immune response towards an anti-inflammatory phenotype. Interestingly, imatinib ameliorated neuroinflammation, even when the treatment was initiated after the clinical manifestation of the disease. We have previously shown that imatinib reduces BBB disruption and stroke volume after experimentally induced ischemic stroke by targeting platelet-derived growth factor receptor -α (PDGFR-α) signaling. Here we demonstrate that PDGFR-α signaling is a central regulator of BBB integrity during neuroinflammation and therefore imatinib should be considered as a potentially effective treatment for MS.

## Introduction

The CNS vasculature is specialized in keeping the CNS tissue in an immune-privileged environment. The blood-brain barrier (BBB), with counterparts found in the spinal cord (BSCB) and retina (BRB), represents both an anatomical and a functional unit mediating molecular transport and immune regulation. The BBB consists of a layer of tightly adhering endothelial cells lining the blood vessel lumen that actively and selectively restrict passage of water, ions, metabolites, and cells. Although the endothelium provides the main physical barrier, tightly associated pericytes and astrocytes contribute to the barrier function, and together with the endothelium, microglia and connecting neurons comprise the neurovascular unit [1].

During neuroinflammation, cytokines produced by pathogenic T-cells, macrophages and resident brain microglia mediate upregulation of adhesion molecules in the BBB [2], [3]. This leads to BBB activation and subsequent breakdown resulting in influx of immune cells into the CNS [4]. The BBB also secretes chemokines and cytokines, thereby additionally stimulating both proliferation and recruitment of inflammatory cells into the CNS [3]. This self-sustaining vicious cycle of neuroinflammation could be well modulated and reduced by targeting the mechanisms regulating BBB function and integrity.

Disruption of the BBB is an early and continuous event in many CNS disorders, including Multiple sclerosis (MS), a neuroinflammatory disease associated with demyelination, axonal loss and brain atrophy [5]. To dissect the genetic and pathological mechanisms of neuroinflammation, several animal models are used. Myelin oligodendrocyte glycoprotein (MOG)-induced experimental autoimmune encephalomyelitis (EAE) in rats is a well-characterized animal model of MS, sharing several important features with the human disease including T helper 1 (Th1) and B-cell involvement, as well as histopathological features [6]. MS is characterized by demyelinated lesions and perivascular cuffs containing primarily T-cells and macrophages, indicating a compromised BBB [7].

Platelet-derived growth factor -CC (PDGF-CC) is a proteolysis-activated growth factor that promotes opening of the BBB [8], [9]. Blocking signaling through its receptor, PDGF receptor-α (PDGFR-α), using the tyrosine kinase inhibitor imatinib mesylate (Gleevec®), reduces BBB disruption and stroke volume after experimental ischemic stroke [9], [10]. Since imatinib has been shown to block PDGFR-α signaling and reduce both cerebrovascular permeability and hemorrhagic complications in two different stroke models [9], [10], we aimed to explore a possible beneficial effect of imatinib in the MS model. In our hands, imatinib treatment significantly enhanced BBB integrity and ameliorated neuroinflammation, and subsequently delayed the disease onset and attenuated the disease course.

## Materials and Methods

### Ethics Statement

All experiments in this study were approved and performed in accordance with the guidelines from the Swedish National Board for Laboratory Animals and the European Community Council Directive (86/609/EEC) under the ethical permit N65/10, which was approved by the North Stockholm Animal Ethics Committee. Rats were tested according to a health-monitoring program at the National Veterinary Institute (SVA) in Uppsala, Sweden.

### EAE Induction, Imatinib Treatment and Clinical Scoring

Inbred DA rats were obtained from Harlan (USA) and C57BL/6 mice were obtained from Charles River (Germany). Rats and mice were housed in the animal facility at the Karolinska Institute and Hospital (Stockholm, Sweden) in a pathogen-free and climate-controlled environment in polystyrene cages containing aspen wood shavings with free access to standard rodent chow and water with regulated 12-hour light/dark cycles. MOG, amino acids 1–125 from the N-terminus used for the rat immunization and 130 amino acids from the extracellular domain of MOG used in mice, was expressed in Escherichia coli and purified to homogeneity by chelate chromatography [11]. The sequence used for mice immunization: MGQFRVIGPGYPIRALVGDEAE LPCRISPGKNATGMEVGWYRSPFSRVVHLYRNGKDQDAEQAPEYRGRTELL KETISEGKVTLRIQNVRFSDEGGYTCFFRDHSYQEEAAMELKVEDPFYWLEHHHHHH. The purified protein, dissolved in 6 M urea, was dialyzed against phosphate buffered saline (PBS) to obtain a physiological preparation that was stored at −80°C. Female rats at the age of 12 weeks were anaesthetized with isoflurane (Forene, Abbott Laboratories, Chicago, IL, USA) and injected subcutaneously in the tail base with a 200 µl inoculum containing 15 µg MOG in PBS, emulsified 1∶1 with incomplete Freund’s adjuvant (Sigma-Aldrich, St. Louis, MO, USA) in order to induce EAE. Similarly, female mice at the age of 12 weeks were anaesthetized with isoflurane and injected subcutaneously in the tail base in order to induce EAE with a 100 µl inoculum containing 50 µg MOG in PBS, emulsified 1∶1 with complete Freund’s adjuvant (incomplete Freund’s adjuvant with 2 mg/ml mycobacterium tuberculosis). The daily dose of 250 mg/kg/animal of imatinib or PBS vehicle were split into a morning (1/3) and 8h later, an evening dose (2/3); administrated via oral gavage. The treatment was initiated either on the day of immunization, day 2 p.i., day 5 p.i., or after clinical onset of EAE (score 1; exact starting point for gavage feeding is indicated in the description of each performed experiment). Imatinib tablets (Novartis; Switzerland) were crushed into a fine powder, solubilized in sterile PBS, vortexed and incubated at 37°C (water bath) for 5 minutes. Insoluble components were spun down in a table microcentrifuge (13000 rpm) for 2 minutes. The supernatant was used immediately for oral gavage performed with soft and steel gavage needles for rats and mice, respectively.

Rats and mice were monitored daily for clinical signs of EAE, from day 10 until the day of sacrifice at day 23–33 p.i. Score 0 = no clinical signs of EAE; 1 = tail paralysis; 2 = hind leg paraparesis or hemiparesis; 3 = hind leg paralysis or hemiparalysis and 4 = tetraplegy or moribund. The following clinical parameters were assessed for each animal: maximum EAE score (the highest clinical score observed during EAE), cumulative EAE score (the sum of daily clinical scores) and duration of EAE (the number of days with EAE).

### Quantitative Real-time PCR and Expression Array Analysis

Draining inguinal lymph nodes were collected on day 10 p.i. and placed in DMEM (Gibco-BRL, Grand Island, NY, USA) before being mechanically separated by passage through a mesh screen with the bolus of a syringe. mRNA was extracted from the single cell suspension using the RNeasy kit (Qiagen, Hilden, Germany) and the QIAcube (Qiagen) including on column DNA-digestion for fully automated sample preparation. RNA concentration and purity was determined through measurement of A260/A280 ratios with a NanoDrop ND-1000 Spectrophotometer (NanoDrop Technologies, Wilmington, DE, USA). Confirmation of RNA quality was assessed using the Agilent 2100 Bioanalyzer (Agilent Technologies, Santa Clara, CA, USA). mRNA was subsequently either used for expression array analysis or cDNA generation for qPCR analysis. cDNA was prepared using the iScript kit (Bio-Rad, Hercules, CA, USA). Quantitative real-time PCR (qPCR) was performed using a CFX384 touch real-time PCR detection system (Bio-Rad) with a three-step PCR protocol (95°C for 10 minutes followed by 40 cycles of 95°C for 10 sec and 60°C for 30 sec and 45 cycles of melt curve analysis), using SYBR Green (Bio-Rad) as the fluorophore. Relative expression levels, corrected for amplification efficiency, were analyzed using the CFX manager software (Bio-Rad). Relative expression was calculated as the ratio between the target and Gapdh. Serial 10-fold dilutions from a pool of undiluted samples within the study were used as standard. The primers used were the following: STAT6_fw AAC AGC AGC TGG CAG GGA ATG G, STAT6_rev GCT TCT CCA CCA GGA AAG AGC TGG, GAPDH_fw TCA ACT ACA TGG TCT ACA TGT TCC AG, GAPDH_rev TCC CAT TCT CAG CCT TGA CTG, CD4_fw GCT CCC ACT CAC CCT TCA GAT AC, CD4_rev CTT CAC CTT CAC TCA GTA GAC ATT GC, TLR2_fw CCA GAG GAC TCA GGA GCA G, TLR2_rev CAC ACA CCA GCA GCA TCA C, IL4_fw CTT ACG GCA ACA AGG AAC ACC, IL4_rev CTT TCA GTG TTG TGA GCG TGG.

Expression array analysis was performed on Affymetrix rat 1.0 ST 3′arrays at the BEA core facility (Huddinge, Stockholm, Sweden). The mean Plier value of each group and expression fold change from those transcripts that were significantly different in the two groups was analyzed and calculated using Ingenuity Pathways Analysis (Ingenuity Systems, CA, USA, www.ingenuity.com). Molecules from the dataset that met the<or >1,3 fold change cutoff and were associated with biological functions and/or diseases or were associated with a canonical pathway in Ingenuity’s knowledge base were considered for the analysis. Right-tailed Fisher’s exact test was used to calculate a p-value determining the probability that each biological function and/or disease assigned to that data set is due to chance alone. The significance of the association between the data set and the canonical pathway was measured in 2 ways: 1) A ratio of the number of molecules from the data set that map to the pathway divided by the total number of molecules that map to the canonical pathway is displayed. 2) Fisher’s exact test was used to calculate a p-value determining the probability that the association between the genes in the dataset and canonical pathway is by chance alone.

### Elispot Analysis and MOG Re-stimulation Assay

For MOG re-stimulation assay and Elispot analysis spleens were harvested on day 7 or 10 p.i., respectively, and placed in DMEM before being mechanically separated by passage through a mesh screen with the bolus of a syringe. For Elispot analysis spleenocytes (150000/well) were stimulated for 48 hours in 96-well plates with Concavalin A (ConA, 0.125 ng/µl), myelin oligodendrocyte protein (MOG, 0.5 ng/µl) or myelin basic protein (MBP, 0.5 ng/µl, here used as unspecific antigen). The IFNγ Elispot (R&D Systems) was performed according to the manufacturer’s instructions. IFNγ production was measured with an AID Elispot reader (AID, Germany).

The MOG re-stimulation assay was performed using the Bioplex Th1/Th2 kit (Bio-Rad, CA, USA) according to the manufacturer’s specifications. 1 million spleenocytes/well were stimulated with ConA, MOG and MBP (respective working concentrations as indicated above) in 48-well plates for 3 days. The concentrations of Th1 and Th2 specific cytokines was measured by the Bioplex 200 system plate reader.

### Histopathological and Immunohistochemical (IHC) Analysis

On day 10 and 14 p.i., respectively, animals were euthanized using CO_2_ and perfused with PBS followed by 4% paraformaldehyde (PFA). Paraffin embedded brain and spinal cord cross-sections (3–5 µm thick) were dewaxed in xylol, rehydrated and then stained with Hematoxylin & Eosin (HE) and Luxol Fast Blue (Kluever) to assess tissue inflammation and demyelination, respectively. The inflammatory index (I.I.) and demyelination score (DM) were determined from the number and size of demyelinated lesions of each animal on an average of ten complete spinal cord cross-sections and brains as previously described [6].

In adjacent serial sections IHC analysis were performed by using antibodies against the following targets: α-CD68 (ED1, mouse anti-rat, AbD Serotec, 1∶1000), CD43 (W3/13 mouse anti-rat, Abcam, 1∶100), α-Dysferlin (Ham1/7B6, mouse anti-rat, Abcam, prediluted), α-Occludin (rabbit anti-rat, Abcam, 1∶100) and CCR2 (rabbit anti-rat, Abcam, 1∶500) diluted in 10% fetal calf serum (FCS) in PBS. Control sections were incubated in the absence of the primary antibody.

For IHC, paraffin sections of the spinal cord were treated as previously described [12]. After deparaffinization in xylol, sections were transferred to 90% ethanol. Endogenous peroxidase was blocked by incubation in methanol with 0.02% H_2_O_2_ for 30 minutes at room temperature (RT) and rehydration to distilled water followed via a 90%, 70%, and 50% ethanol series. Antigen retrieval was performed with ethylenediamine tetraacetic acid buffer, pH 8.5, or citrate buffer pH 6.0 by warming for 1 hour in a steamer device (Braun, Germany). Sections were incubated in 10% FCS in 0.1 M PBS 30 minutes prior to incubation with primary antibody on 4°C, over night. After washing in PBS, sections were incubated with biotinylated secondary antibody (biotinylated anti-mouse or rabbit; Amersham Pharmacia Biotech, 1∶200) for 1 hour at RT. All stainings were performed with biotin-avidin peroxidase detection system and visualized with 3,3′diaminobenzidine-tetrahydrochloride (DAB, Sigma). Evaluation was performed on at least ten whole spinal cord cross-sections per animal by using Leica Polyvar 2 microscope.

Toluidine blue staining was performed on mouse lymph node and spleen tissue harvested on day 7 p.i., as well as on rat spinal cord harvested on day 14 p.i. Tissues were immersion-fixed with PFA over night at 4°C, cryo-protected in 20% sucrose, embedded and cryo-sectioned. Sections mounted on pre-adhesive glass slides were incubated in a solution containing 0.5% Toluidine blue (w/v) in 1% NaCl, pH 2.3 (Sigma) for 3 minutes. The staining was captured using an inverted microscope (Axio Observer, Zeiss, Germany). Counted mast cells are presented as cell number/mm^2^ (individual observations based on 8 cross-sections). Surface areas of evaluated tissue were measured by Axio-vision software (Zeiss).

### Tracer Injection and Immunofluorescence (IF) Staining Procedures

Under isoflurane anesthesia, 25 mg (0.5 ml) dextran-TMR (70 kDa, Invitrogen) was intravenously infused at day 10 and day 14 p.i., respectively. The tracer was allowed to circulate for 2 hours and thereafter the animals were transcardially perfused with HBSS followed by 4% PFA. Spinal cords were immediately removed and post-fixed for 2 hours in PFA and thereafter cryo-protected in 20% sucrose, 4°C o/n. Individual spinal cords were photographed before being embedded. To obtain sections from different segments, spinal cords were first cut in 7 mm long pieces, rostral to caudal. Cryosections of 12 µm thickness from the different spinal cord segments were obtained and IF procedures were performed as described below.

Mouse monoclonal antibodies against CD11b (Ox42), CD68 (ED1), MHC class II (Ox-6), CD43 (W3/13), CD8 and CD45L (Ox-22) were purchased from AbD Serotec (Germany). Mouse monoclonal anti-CCR2, rabbit polyclonal anti-CD3 and mouse monoclonal anti-tryptase antibodies were obtained from Abcam and mouse monoclonal anti-CD45RA antibody was purchased from eBioscience (Austria). Sections were air-dried for 30 minutes before permeabilization in PBS/0,2% Triton-x100 for 10 minutes. Staining against MHC class II (Ox-6), CD3 and CD8 required antigen retrieval with citrate buffer pH 6.0.

Blocking was performed in TNB buffer (Perkin Elmer) and primary antibodies diluted in TNB buffer were applied over night at 4°C at a dilution of 1∶200. The antibody signal was visualized using Alexa-Fluor conjugated secondary antibodies (Invitrogen).

Dextran and IF images were captured using a confocal microscope (Zeiss LSM700). Representative images shown are 2D renderings of 10 µm thick z-stacks. Fluorescence quantifications (pixel area) were performed using Image J software (NIH). The individual observations are based on analysis of five fields of vision from comparable anatomic positions.

### Isolation of Endothelial Vessel Fragments and qPCR Analysis

Imatinib or PBS treated mice were anesthetized at day 13 p.i. with hypnorm and midazolam and thereafter transcardially perfused with HBSS. Subsequently, spinal cords were rapidly dissected and placed into ice-cold HBSS. The rest of the protocol was performed as described, except for the antibody and magnetic beads for pulling out the endothelial vessel fragments (EVF) [13]. We used biotinylated rat anti-mouse CD31 antibody (BD Biosciences) together with magnetic beads (Dynabeads biotin binder, Invitrogen Dynal AS, Oslo, Norway). mRNA and cDNA were generated as described above (quantitative real-time PCR). Real-Time qPCR was performed using KAPA SYBR FAST qPCR Kit Master Mix (2x) Universal (KAPA Biosystems) in Rotor-Gene Q (Qiagen) Real-Time PCR thermal cycler according to the manufacturers’ instructions. Expression levels were normalized to the expression of L19. The following primers were used: VCAM-1_fw TGC CGA GCT AAA TTA CAC ATT G, VCAM-1_rev CCT TGT GGA GGG ATG TAC AGA, P-selectin_fw CCT GGC AAG TGG AAT GAT GA, P-selectin_rev AAG CTG CAG ACT GAC TGG TA, ICAM-1_fw GCT TTG AGA ACT GTG GCA CC, ICAM-1_rev TGA GGT CCT TGC CTA CTT GC, CD74_fw CCA TGG ATG GCG TGA ACT GG, CD74_rev ATG TGG CTG ACT TCT TCC TGG, CCL19_fw ATG TGA ATC ACT CTG GCC CAG GAA, CCL19_rev AAG CGG CTT TAT TGG AAG CTC TGC, CCL2_fw TTA AAA ACC TGG ATC GGA ACC AA, CCL2_rev GCA TTA GCT TCA GAT TTA CGG GT, CXCL2_fw GGC AAG GCT AAC TGA CCT GG, and CXCL2_rev AGG CAC ATC AGG TAC GAT CC.

To analyze the recruitment of the naive T-cells, local draining lymph nodes were harvested on day 2 p.i. mRNA and cDNA was isolated and qPCR analysis performed as described above. The following primers were used: L-selectin_rev CTT CAC GGG AGG ACT TGA CG, L-selectin_fw TTC TCA TTT GGC TGG CAA GG, Glycam1_rev CTC ACT GGT GTA GCT GGT GG, Glycam1_fw CAC CTC TCT TGC TCT CCT GC, CCR7_rev ATC GGT GAC CTC ATC TTG GC, CCR7_fw CAG GAA AAA CGT GCT GGT GG, CD34_fw AGC AGT AAG ACC ACA CCA GC, CD34_rev CCA GTT GGG GAA GTC TGT GG.

### Statistical Analysis

The results from all experimental analysis, except for EAE scores are depicted as average ± S.E.M. from the number of observations stated in the figure legends. Statistical evaluation was performed using non-paired Student’s t-test with statistical significance defined as *P≤0.05, **P≤0.01 and ***P≤0.001. EAE scores are depicted as mean ± S.E.M. from the number of observations stated in the figure legends. Statistical evaluation was performed using Kruskal-Wallis test with statistical significance defined as *P≤0.05, **P≤0.01 and ***P≤0.001.

## Results

### Imatinib Enhances BBB Integrity

As imatinib is known to reduce brain edema and hemorrhage after stroke by inhibiting the PDGF-CC pathway and thus improving BBB integrity [9], [10], we hypothesized that imatinib may also affect the BBB during neuroinflammation. For that purpose we immunized DA rats with MOG/IFA to induce EAE. Animals were split into two groups and treated by oral gavage either with imatinib or with PBS, as a mock control. Treatment was initiated 5 days post immunization (p.i.), before BBB breakdown and the clinical manifestation of the disease has been observed.

**Figure 1:**
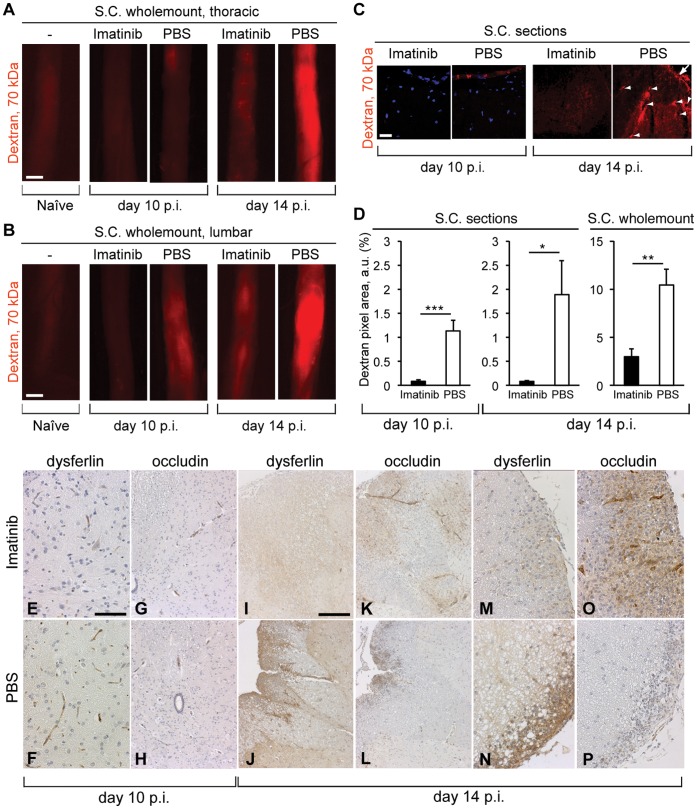
Imatinib enhances BBB integrity during EAE. (A, B) Extravasation of 70 kDa dextran in wholemount thoracic and lumbar spinal cord portions on day 10 and 14 p.i. Imatinib-treated rats exhibited less extravasation on both time-points, compared to PBS controls. Tracer-injected naive rat is shown as negative control. (C) Dextran extravasation in spinal cord cross-sections. PBS-treated rats showed distinct meningeal permeability on day 10 p.i., which was not observed in the imatinib-treated animals. On day 14 p.i., imatinib-treated rats showed less signs of BBB disruption in contrast to profound extravasation in the meninges and perivascular space in both gray and white matter of the control spinal cords (white arrows). (D) Quantification of vascular permeability on day 10 and 14 p.i. based on red fluorescent pixel area recorded in spinal cord sections and wholemounts (n = 5 rats/experimental group/time-point). Scale bars, 1 mm (A, B) and 50 µm (C). Error bars, S.E.M. Statistics were calculated using t-test and *P* values <0.05 were considered significant. *P*<0.05 = *, *P*<0.01 = **, *P*<0.001 = ***. IHC analysis of paraffin embedded spinal cord tissue sections on day 10 p.i. (E–H) and 14 p.i. (I–P). α-dysferlin was used for detecting permeable CNS vasculature and α-occludin for detecting the tight junction components. (E–H) Healthy animals from both groups were compared on day 10 p.i. (n = 8 rats/experimental group; representative images shown). Almost total absence of dysferlin^+^ blood vessels observed in the spinal cord gray matter of the imatinib group (E), while PBS controls exhibited dysferlin^+^ vessels more frequently (F). Occludin^+^ blood vessels were rarely detectable in both groups (G, H). (I–P) On day 14 p.i. (n = 5 rats/experimental group; representative images shown), spinal cord tissues from the same anatomical positions undergoing EAE from both groups were compared. Demyelinated lesions and lesion-associated blood vessels in the imatinib-treated rats expressed predominantly occludin (K, O), while dysferlin upregulation prevailed in the lesions of the PBS controls (J, N). Scale bars, 100 µm (E–H, M–P), 250 µm (I–L). Imatinib or PBS oral gavage was performed from day 5 p.i until the end of the experiment (A–P).

The integrity of the BBB was assessed on day 10 and day 14 p.i. by injecting fluorescently labeled 70 kDa dextran into the tail vein. BBB integrity was evaluated according to the degree of tracer extravasation into the perivascular tissue. Visualization and quantification of spinal cord cross sections and whole-mount preparations demonstrated enhanced BBB integrity in the imatinib-treated rats compared to PBS controls on both day 10 and 14 p.i. (Fig. 1 A–C). On day 10 p.i., PBS controls showed distinct meningeal permeability for the tracer which was not observed in the imatinib group. On day 14 p.i., imatinib-treated rats exhibited less BBB disruption compared to profound subpial and perivascular extravasation of the tracer observed in the spinal cord tissue of the PBS controls. Quantification of affected areas performed on the spinal cord whole-mount preparations and sections demonstrated direct correlations between imatinib treatment and enhanced BBB integrity, on both day 10 and day 14 p.i. (Fig. 1 D).

BBB integrity was also assessed by immunohistochemical (IHC) analysis of the spinal cord tissue on day 10 p.i. and 14 p.i. (Fig. 1 E–P). As a read-out we used dysferlin, a marker for leaky brain vasculature, which also reveals dissociation of perivascular inflammatory infiltrates and BBB disturbance in MS, and occludin, a marker detecting one of the main BBB tight junction components [14], [15]. On day 10 p.i., the imatinib group showed no signs of CNS inflammation, while 50% of the control group already developed demyelinated lesions in the brain and/or spinal cord. When we compared healthy animals from both groups, we observed almost total absence of dysferlin^+^ blood vessels in the spinal cord gray matter of the imatinib-treated rats, while PBS controls exhibited dysferlin^+^ blood vessels more frequently (Fig. 1 E, F). At the same time-point, occludin^+^ blood vessels were rarely detectable in both groups (Fig. 1 G, H). IHC analysis of the PBS controls which have developed neuroinflammation on day 10 p.i., revealed upregulation of dysferlin in the spinal cord white matter lesions and lesion associated blood vessels (data not shown). On day 14 p.i., disease incidence and severity continued to be higher in the control group (100% vs. 40%). When comparing spinal cord tissue undergoing EAE from both groups, we observed a shift in the dysferlin-occludin expression ratio within the same lesions. Demyelinated lesions and lesion-associated blood vessels in the imatinib-treated rats expressed predominantly occludin, whereas in PBS controls dysferlin upregulation prevailed (Fig. 1 I–P). Besides upregulation in the white matter lesions, massive leaky dysferlin^+^ lesion associated blood vessels were occasionally observed in the PBS control group white matter, but not in the imatinib-treated group. This data additionally supports our observation of a better preservation of BBB integrity in response to imatinib treatment.

### Imatinib Reduces Cell Recruitment to the Inflammation Site

To compare the effects of imatinib treatment and PBS vehicle control in the CNS undergoing EAE, we performed histopathological, as well as extensive IHC and immunofluorescence (IF) analysis on spinal cord transverse sections, including quantitative evaluation performed on dextran-perfused spinal cords.

**Figure 2:**
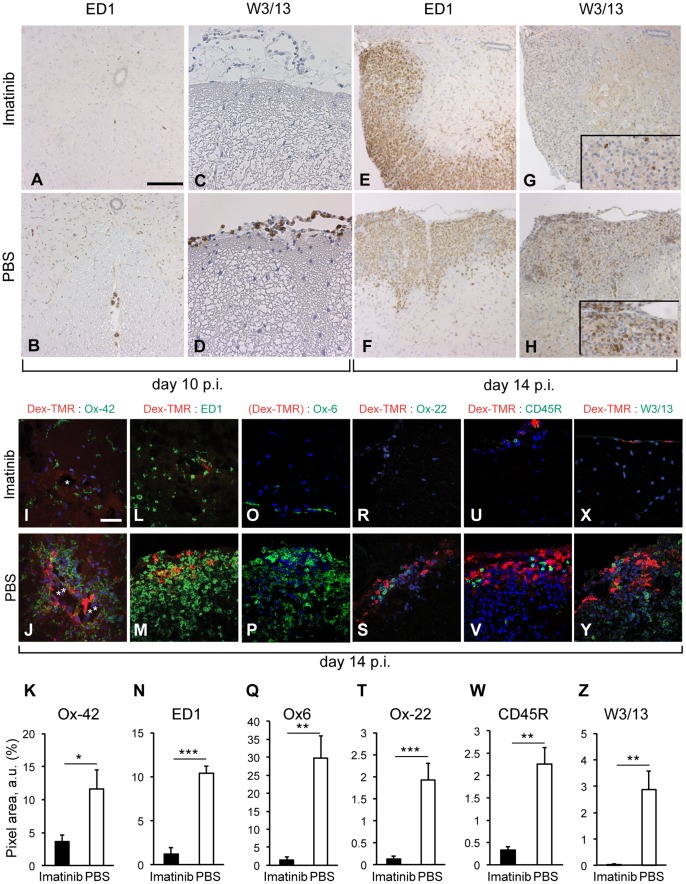
Imatinib reduces infiltration of immune cells and attenuates microglia activation. IHC analysis on paraffin embedded spinal cord tissue cross-sections on day 10 p.i. (A–D; n = 8 rats/experimental group; representative images shown) and 14 p.i. (E–H; n = 5 rats/experimental group; representative images shown). Although showing no sign of CNS inflammation and demyelination, control animals already started recruiting W3/13^+^ T-cells (B) and ED1^+^ macrophages (D) to the meninges and in the perivascular space, whereas the imatinib-treated group showed a delay in recruitment of inflammatory cells to the CNS. On day 14 p.i., spinal cords of the imatinib-treated rats exhibiting demyelinated lesions recruited lower amounts of W3/13^+^ T-cells (G, H) while ED1^+^ macrophages infiltration was similar to the controls (E, F). Scale bar, 200 µm (A–H). (I–Z) IF performed on spinal cord cross-sections of rats injected with fluorescent tracer (dextran, red) on day 14 p.i. α-Ox-42, ED1, Ox-6, Ox-22, CD45RA and W3/13 antibody staining (all in green) in imatinib- (I, L, O, R, U, X) and PBS-treated rats (J, M, P, S, V, Y). (I–K) Microglia activation was significantly decreased in the imatinib-treated rats, while Ox-42^+^ cells were detectable around leaky blood vessels (asterix) in the control tissue. (L–N) The amount of macrophages/activated microglia cells was significantly decreased in the spinal cords of the imatinib-treated rats. (O–Q) Significantly lower amounts of MHC class II^+^ cells were found in the meninges and parenchyma of the imatinib-treated rats vs. PBS controls. (R–Z) Significantly lower amounts of Ox-22^+^, CD45RA^+^ and W3/13^+^ cells were found in the meninges and parenchyma of the imatinib-treated rats vs. PBS controls (R, U, X vs. S, V, Y). Quantifications of Ox-42, ED1, Ox-6, Ox-22, CD45RA and W3/13 expression based on green fluorescent pixel area quantifications from spinal cord cross-sections (K, N, Q, T, W, Z). n = 5 rats/experimental group. Scale bar, 50 µm. Error bars, S.E.M. Statistics were calculated using t-test and *P* values <0.05 were considered significant (*P*<0.05 = *, *P*<0.01 = **, *P*<0.001 = ***). Imatinib or PBS oral gavage was performed from day 5 p.i until the end of the experiment.

On day 10 p.i., we observed a delay in recruitment of inflammatory cells in the imatinib-treated group (Fig. 2 A, C) in contrast to the control tissue which already started recruiting T-cells and macrophages to the meninges and in the perivascular space (Fig. 2 B, D). Here we compared CNS tissue lacking signs of demyelination from both groups, as the imatinib-treated group at this time-point had not yet developed the disease. This data indicate an earlier disease onset in the PBS compared to the imatinib-treated group.

Notably, extensive IHC/IF analysis on day 14 p.i., revealed that spinal cords from imatinib-treated rats undergoing neuroinflammation tend to recruit a lower amount of W3/13^+^ T-cells to the demyelinated lesions, while ED1^+^ macrophage infiltration was similar to control rats exhibiting comparable inflammatory index and demyelination score (Fig. 2 E–H). Inflammatory cells and activated CNS resident microglia were commonly localized in areas exhibiting dextran extravasation (meninges/perivascular space), while it seemed that imatinib partially prevented the entry of T-cells into the brain parenchyma (Fig. 2 I–Z). Finally, quantitative evaluations in correlation with dextran extravasation demonstrated generally significantly lower amount of recruited inflammatory cells in the imatinib-treated rats (Fig. 2 K, N, Q, T, W, Z). Moreover, immunostaining for Ox-22, CD45RA and Ox-42 (CD11b/c) revealed almost complete absence of cytotoxic T- and B-cells as well as lower monocyte chemotaxis in the imatinib-treated group. On the other hand, screening for the mast cell density performed by toluidine blue staining (Fig. S1) as well as by IF staining against mast cell tryptase (data not shown) revealed no significant difference between imatinib treatment and PBS in EAE. Analysis revealed comparable amounts of mast cells in both experimental groups in lymph nodes and spleen day 7 p.i as well as in the spinal cord day 14 p.i (Fig. S1, data not shown).

### Imatinib Shifts the Immune Response and Prohibits Activation of MOG Specific T-cells

**Figure 3:**
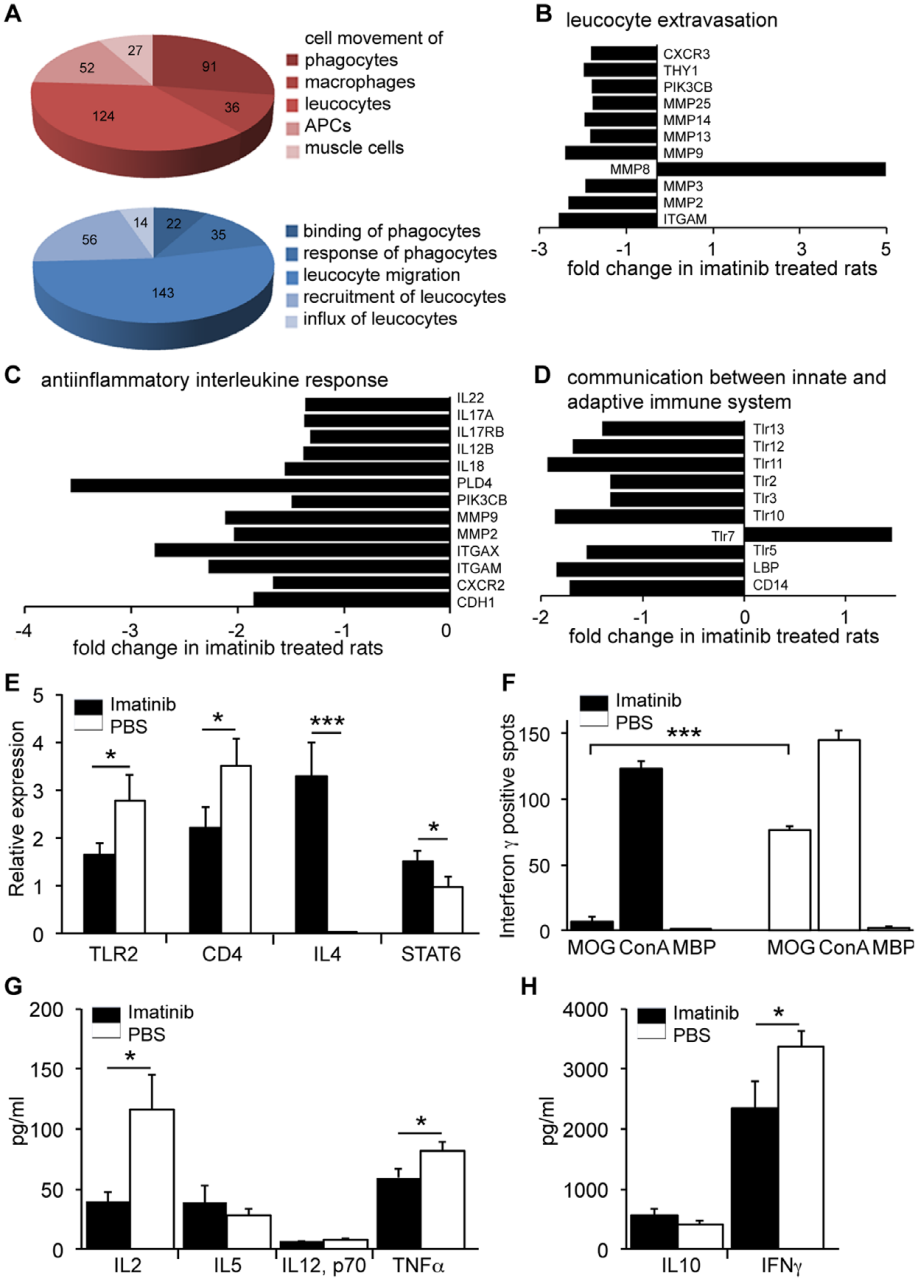
Imatinib suppresses the peripheral immune response. (A–D) Genome wide expression array analysis performed on inguinal lymph node cells harvested from imatinib-treated and control rats on day 10 p.i. (Affymetrix 1.0 ST. 3' arrays; n = 6 arrays/experimental group). (A) Functional annotations differentially regulated between imatinib and PBS-treated rats. Immune cell trafficking was profoundly downregulated in the imatinib group (red pie-chart), as well as numerous immune functions (blue pie-chart). Numbers indicate the amount of molecules differentially expressed in the certain biological function. (B–D) Canonical pathways most significantly affected by imatinib treatment. Leucocyte extravasation was downregulated in the imatinib-treated rats, especially matrix metalloproteinases and CXCR3 (B). The anti-inflammatory interleukine response is also downregulated in the imatinib group in contrast to controls (C). The communication between the innate and adaptive immune response, especially Toll-like receptor (Tlr) signaling is generally downregulated in the imatinib group (D). Statistics are calculated using t-test and calculated *P* values indicated high significance for each presented molecule *P*<0.00001 = ***). Error bars (not visible), S.E.M. (E) Gene expression profiling in inguinal lymph nodes day 10 p.i. by qPCR. mRNA transcript levels for Th2-cell lineage proliferation: *IL4* and *STAT6* are higher in imatinib-treated rats., whereas control rats showed elevated mRNA levels for *TLR2* and *CD4* transcripts (n = 8 rats/experimental group, both inguinal lymph nodes/animal). (F) MOG-induced IFNγ Elispot analysis on imatinib-treated and control rat spleenocytes harvested on day 10 p.i. ConA used as a positive control, MBP as an unspecific antigen (n = 4 rats/experimental group). Imatinib-treated rats had significantly lower number of proliferating MOG specific T-cells comparing to the controls. (G–H) MOG re-stimulation assay with spleenocytes harvested from imatinib-treated or control mice on day 7 p.i. (n = 4 mice/experimental group). Levels of Th1/Th2 specific cytokines measured after three days *in vitro* culturing in the presence of MOG, MBP or ConA. (A–F) Imatinib or PBS oral gavage was performed from day 5 p.i until the end of the experiment. (G–H) Imatinib or PBS oral gavage was performed from day 2 p.i until the end of the experiment Error bars, S.E.M. Statistics were calculated using t-test and *P* values <0.05 were considered significant (*P*<0.05 = *, *P*<0.01 = **, *P*<0.001 = ***).

**Figure 4:**
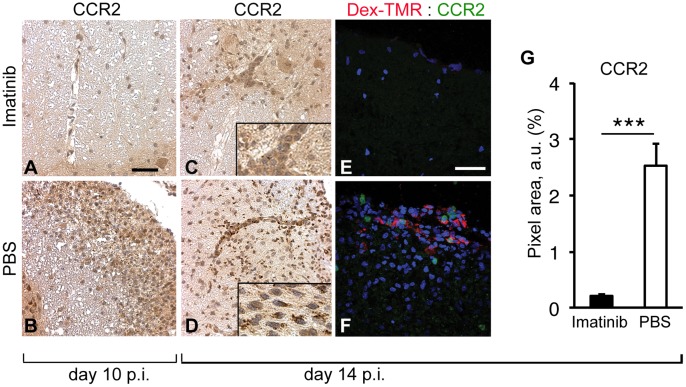
Imatinib downregulates CCR2 expression in the CNS during EAE. (A–D) IHC analysis of CCR2 expression (in brown) performed on paraffin embedded spinal cord cross-sections on day 10 p.i. (n = 8 rats/experimental group) and 14 p.i. (n = 5 rats/experimental group). On day 10 p.i. CCR2^+^ cells were clearly detectable in the lesions of the PBS rats (B), while they were not present in the imatinib group (A). On day 14 p.i., in contrast to the imatinib-treated, spinal cord tissue from the PBS group undergoing EAE harbored high amounts of CCR2^+^ cells located in the blood vessels and perivascularly. Scale bar, 150 µm (A–D). (E, F) IF performed on spinal cord tissue cross-sections of the rats injected with fluorescent tracer (dextran, red) on day 14 p.i. (n = 5 rats/experimental group; representative images shown). Visualization of CCR2 antibody staining (in green), nuclei visualization by DAPI (blue). CCR2^+^ cells here shown in the subpial region exhibiting dextran extravasation and pronounced cell infiltration in the PBS group vs. imatinib-treated spinal cord region with moderate cell infiltration lacking in CCR2^+^ cells (E). (G) IF analysis quantification in correlation with dextran extravasation. CNS of the imatinib-treated rats significantly downregulated CCR2 expression comparing to controls on day 14 p.i. Scale bar, 50 µm (E, F). Imatinib or PBS oral gavage was performed from day 5 p.i until the end of the experiment. Error bars, S.E.M. Statistics were calculated using the t-test and *P* values <0.05 were considered significant (*P*<0.05 = *, *P*<0.01 = **, *P*<0.001 = ***).

In order to avoid biased assessment of pathways/biological protective effects of the imatinib treatment in EAE, we performed genome wide expression analysis using Affymetrix rat 1.0 ST 3′arrays. mRNA isolated from both inguinal lymph nodes on day 10 p.i. of individual rats treated with imatinib or PBS, respectively, was used for hybridizing onto the arrays. Leucocyte cell movement, recruitment and influx, as well as migration of antigen presenting cells (APCs) appeared affected and all significantly downregulated in the imatinib-treated rats (Fig. 3 A). Chemokines and chemokine receptors involved in leucocyte and APC recruitment such as *CCL9, CXCR3, CX3CR1, CXCR2, CCR5 and CCR2* were significantly downregulated in imatinib-treated rats (Table 1). Notably, *CCR2* was downregulated 4-fold in response to imatinib treatment. It has been shown that CC chemokine receptor (CCR) 2 is crucial for EAE pathogenesis. Intriguingly, CCR2 knockouts are resistant to EAE [16], [17]. Therefore, we aimed to investigate whether the decreased amount of infiltrating immune cells in the imatinib-treated rats was related to downregulation of CCR2 in the target organ of EAE. Indeed, according to IHC/IF analysis of the spinal cord on day 10 and 14 p.i. (the quantification of IF staining performed in correlation with dextran extravasation) the CNS of imatinib-treated rats expressed CCR2 far less than the controls (Fig. 4). Moreover, *CCL11,* a chemokine known to attract Th2 cells was upregulated more than 3-fold in the imatinib group (Table 1) [18]. Recently we showed that genetically increased CCL11 expression leads to a milder EAE disease course [19]. Further data analysis elucidated that both leucocyte extravasation and anti-inflammatory interleukin response were significantly downregulated in response to imatinib treatment (Fig. 3 B, C). Both IL17 and its receptor, known to be necessary for pathogenic T-cell recruitment during EAE, were downregulated in the imatinib group (Fig. 3 C). In addition, downregulation of Toll-like receptors in the imatinib-treated group indicated modulated communication between innate and adaptive immunity, resulting in a delayed disease onset (Fig. 3 D). Our data suggest that imatinib treatment modulates the peripheral immune response by stimulating higher production of anti-inflammatory mediators and by reducing the secretion of chemokines involved in leucocyte and APC recruitment.

**Table 1:** Differentially expressed genes in imatinib-treated rats involved in leucocyte and antigen presenting cell (APC) migration and chemotaxis.

gene symbol	gene name	fold change	pvalue	entrez gene	affymetrix ID
CCR2	chemokine (C-C motif) receptor 2	−3.964	0.00001	60463	10914614
CSF1R	colony stimulating factor 1 receptor	−3.032	0.00015	307403	10802065
Mcpt1	mast cell protease 1	−2.974	0.00681	691620	10783998
XCR1	chemokine (C motif) receptor 1	−2.864	0.00010	301086	10921159
ITGAX	integrin, alpha X	−2.779	0.00003	499271	10711299
CCR5	chemokine (C-C motif) receptor 5	−2.679	0.00002	117029	10914618
CX3CR1	chemokine (C-X3-C motif) receptor 1	−2.493	0.00000	171056	10920981
F13A1	coagulation factor XIII, A1 polypeptide	−2.413	0.00009	60327	10794734
PAK1	p21 protein-activated kinase 1	−2.399	0.00087	29431	10708834
CFH	complement factor H	−2.329	0.00000	155012	10768269
ITGAM	integrin, alpha M	−2.271	0.00095	25021	10711268
MMP9	matrix metallopeptidase 9	−2.121	0.00065	81687	10842239
PTAFR	platelet-activating factor receptor	−2.037	0.00079	58949	10880404
MMP2	matrix metallopeptidase 2	−2.036	0.00272	81686	10809540
CCL9	chemokine (C-C motif) ligand 9	−1.990	0.00033	360579	10745662
COL1A1	alpha-1 type I collagen	−1.973	0.02293	29393	10737532
CXCR2	C-X-C chemokine receptor type 2	−1.700	0.00013	29385	10924245
CXCR3	C-X-C chemokine receptor type 3	−1.500	0.00320	84475	10938703
EDN1	endothelin 1	2.350	0.00200	24323	10797857
CXCL3	chemokine (C-X-C motif) ligand 3	2.365	0.00006	114105	10775896
CCL11	chemokine (C-C motif) ligand 11	3.338	0.00000	29397	10736706

In order to confirm whether imatinib treatment was associated with differential T-cell activation, we analyzed the mRNA expression levels of cytokines and transcription factors in the inguinal lymph nodes. On day 10 p.i., *IL-4,* one of the key mediators of the Th2-like response was upregulated in imatinib-, but not in PBS-treated animals. The imatinib group also showed upregulation of the Th2 key transcription factor, *STAT6*. Interestingly, *CD4* and *TLR2* were both upregulated in the PBS group (Fig. 3 E), supporting our observation of an earlier immune response in the control animals.

To see whether response to the specific antigen MOG is altered in imatinib-treated rats, we performed an IFNγ Elispot analysis on *ex vivo* rat spleenocytes harvested on day 10 p.i. (Fig. 3 F). We measured 60% reduced IFNγ production in the imatinib-treated compared to the control group. Importantly, the response to ConA (positive control) and MBP (unspecific antigen) was similar in both groups. These results indicate that imatinib downregulated IFNγ secretion in MOG specific T-cells and thus supported our previous notion that imatinib skews the immune response from Th1 towards Th2 phenotype. In addition, imatinib suppressed the specific T-cell response to MOG by inhibiting clonal T-cell expansion and thereby ameliorated the autoimmune response. T-cell cytokine secretion profile was further assessed through MOG re-stimulated assay. For that purpose, spleenocytes from imatinib- and PBS-treated mice harvested on day 7 p.i., were cultivated for the next 3 days in the presence of MOG, MBP or ConA. Cell culture supernatant of the control spleenocytes contained significantly higher levels of Th1 cytokines (IL-2, TNF-α and IFNγ) compared to those derived from imatinib-treated rats. Th2 specific interleukins, IL-10 and IL-5, correspondingly showed a trend towards increase in the supernatant of the imatinib-treated rats spleenocytes (Fig. 3 G–H).

**Figure 5:**
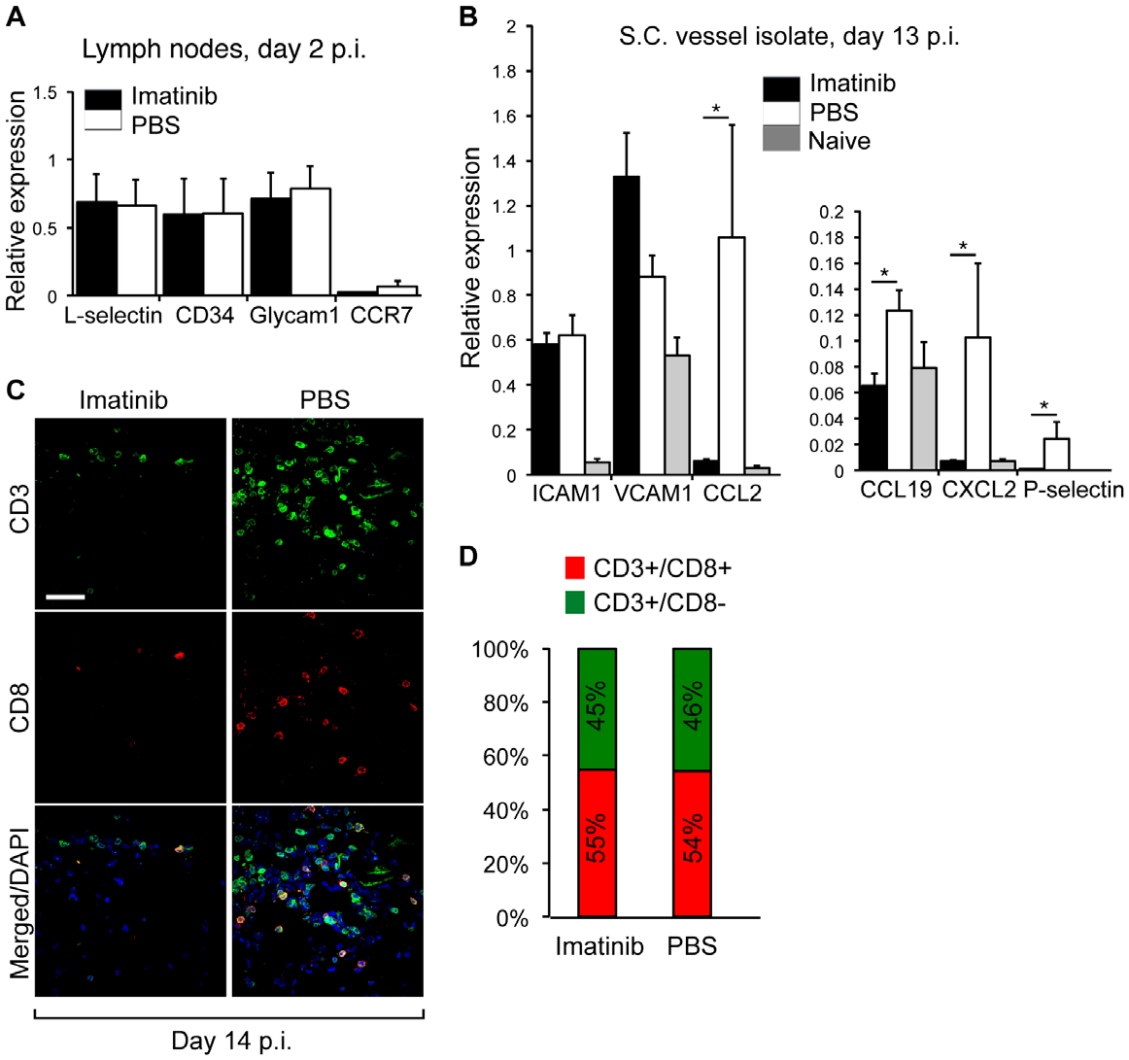
Imatinib inhibits migration of T-cells into the CNS by downregulating chemokine expression in endothelial cells and not by altering recruitment of naïve T-cells into draining lymph nodes. (A) Gene expression profiling in inguinal lymph nodes day 2 p.i. by qPCR. Neither mRNA transcript levels of CD34 and Glycam-1, adhesion markers expressed at the HEV nor CCR7 and L-selectin, homing markers on naïve T-cells were differentially expressed in imatinib vs. control treated mice (n = 4 mice/experimental group, both inguinal lymph nodes/animal). Imatinib or PBS oral gavage was performed from the immunization day until the end of the experiment (day 2 p.i.). (B) Endothelial vessel fragments (EVF) were biochemically isolated from the spinal cord of imatinib or PBS treated mice day 13 p.i. Gene expression profiling by qPCR revealed that P-selectin, CCL2, CCL19 and CXCL2 but not VCAM-1 and ICAM-1 were downregulated in imatinib-treated mice (n = 5 mice/experimental group). Imatinib or PBS oral gavage was performed from day 2 p.i. until day 13 p.i. (C–D). Evaluation of different T-cell subsets in spinal cords from imatinib-treated and control rats revealed less overall infiltration of both CD3^+^/CD8^+^ as well as CD3^+^/CD8^−^ cells in response to imatinib. The relative proportion of CD3^+^/CD8^+^ and CD3^+^/CD8^−^ cells was equal between the treatments. Scale bar, 50 µm. Imatinib or PBS oral gavage was performed from day 5 p.i until the end of the experiment. Error bars, S.E.M., Statistics were calculated using t-test and *P* values <0.05 were considered significant (*P*<0.05 = *, *P*<0.01 = **, *P*<0.001 = ***).

To assess whether imatinib is able to influence the initial T-cell homing into the lymph nodes, imatinib treatment was initiated already on the day of immunization and performed regularly for the next 2 days. Inguinal lymph nodes were harvested on day 2 p.i. and screened for potential differential expression of the markers for the T-cell homing as well as their ligands on the high endothelial venule (HEV). qPCR analysis revealed that neither adhesion markers CD34 and Glycam-1expressed at the HEV, nor homing markers on naïve T-cells, CCR7 and L-selectin, were differentially expressed on the mRNA level in imatinib-treated versus control group (Fig. 5 A). In contrast to expression profiles of the T-cells receptors/ligands observed 2 days p.i., analysis performed on the lymph nodes harvested 10 days p.i. revealed a downregulation of CCR5 (−2.7) and CXCR3 (−1.5), both receptors expressed on activated T-cells important for extravasation of T-cells from the lymph nodes (Table 1).

To analyze whether imatinib influences the expression of adhesion molecules on endothelial cells, EVF were biochemically isolated from spinal cords of imatinib-treated and control mice. Vascular fragments contained the main components of the BBB, namely endothelial cells (200% fold enrichment), pericytes (10% enrichment) and astrocyte end-feet (2% enrichment). EVF were purified by using an antibody directed against CD31. Therefore, a potential contamination with T-cells or macrophages was assessed by qPCR. However, there was no enrichment of immune cells in the vessel isolates (data not shown). Endothelial isolates from imatinib-treated mice exhibited decreased mRNA expression of CCL2, CCL19, CXCL2 and P-selectin, but comparable ICAM-1 and VCAM-1 expression to the control animals (Fig. 5 B). Moreover, detailed IHC/IF analysis performed in spinal cord on day 14 p.i. revealed that both CD3+/CD8+ (CD8) and CD3+/CD8- (CD4) cells were equally reduced in response to imatinib treatment (Fig. 5 C). Although in general less abundant in the imatinib-treated group, CD8 and CD4 T-cell subsets had a similar relative distribution ratio compared to the control-treated group (Fig. 5 D).

### Imatinib Ameliorates Neuroinflammation

**Figure 6:**
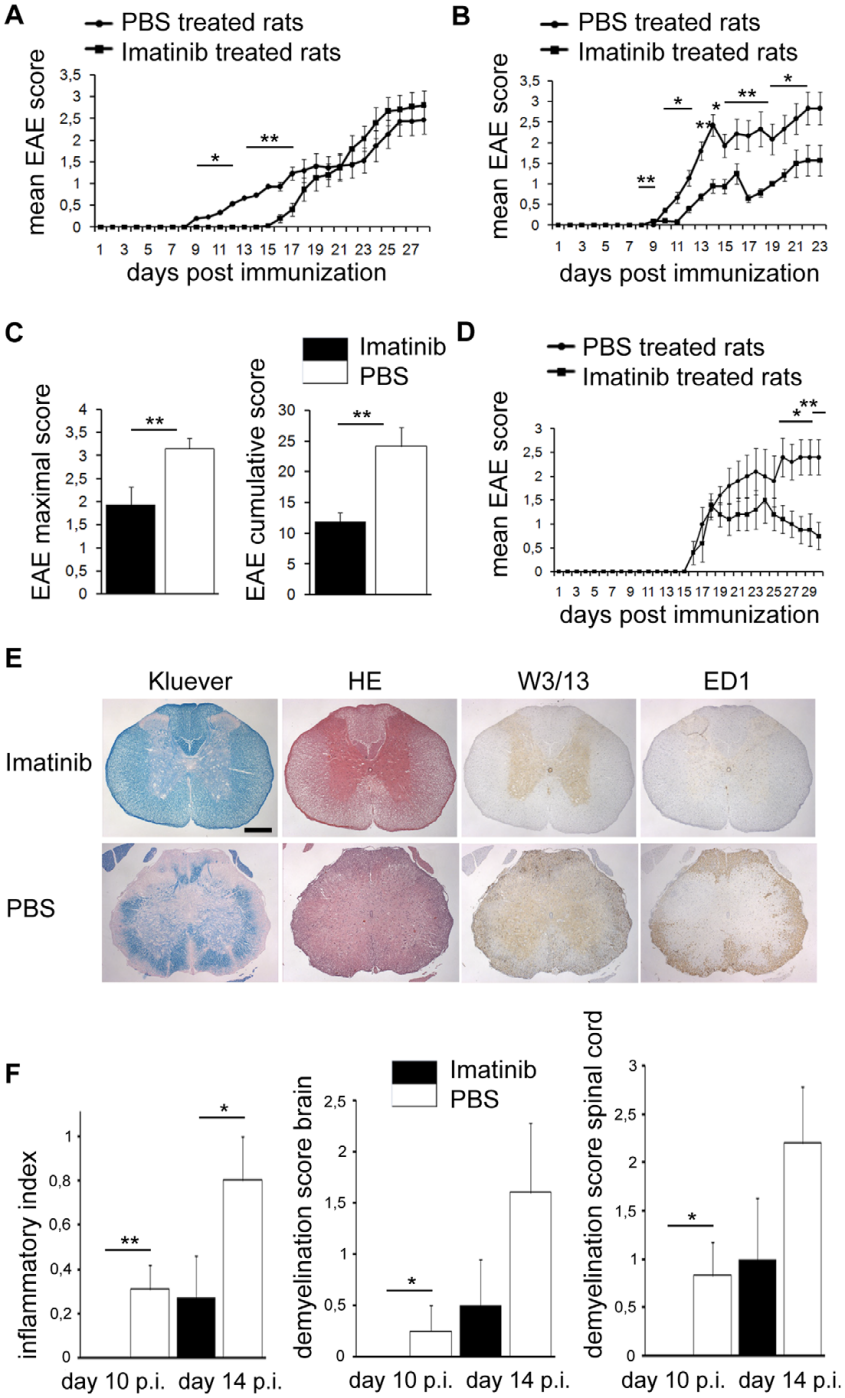
Therapeutic effect of imatinib in EAE. Imatinib-treated rats developed less severe EAE compared to PBS controls. Female rats (n = 15 in each group) treated either with imatinib or PBS from day 5 p.i. until day 10 p.i. (A), or until the end of the experiment (B), respectively. (C) Imatinib-treated rats exhibited lower maximal and cumulative EAE score compared to the control rats (data from the experiment shown in B). (D) Female rats (n = 5 in each group) treated with either imatinib or PBS starting one day after the clinical manifestation of the disease (EAE score 1) until the end of the experiment. Imatinib treatment significantly ameliorated the disease in contrast to the control group. (E) Group representative paraffin embedded spinal cord cross-sections from imatinib and PBS treated rats on day 10 p.i. stained with Kluever (column 1), Hematoxylin-eosin (HE; column 2), W3/13 (column 3), and ED1 (column 4), respectively. Images from the staining performed on day 14 p.i. here not shown. On 10 days p.i. imatinib-treated rats had no signs of CNS inflammation and demyelination, whereas control rats exhibited severe loss of myelin and cell infiltration in the spinal cord white matter lesions, accompanied by recruitment of ED1^+^ macrophages and W3/13^+^ T-cells. Scale bar, 800 µm (F). Graphical representation of the inflammation parameters calculated as inflammatory index (I.I.) and demyelination score (DM) for both brain and spinal cord, respectively, on day 10 (n = 8 rats in each group) and day 14 p.i. (n = 5 rats in each group). Error bars represent S.E.M., statistics were calculated using the t-test and *P* values <0.05 were considered significant (*P*<0.05 = *, *P*<0.005 = **, *P*<0.0005 = ***).

We sought to investigate the effect of imatinib on the clinically manifested neuroinflammation. We hypothesized that an enhanced BBB integrity and reduced recruitment of inflammatory cells in imatinib-treated rats may render the animals less susceptible to the disease. In the first experiment imatinib or PBS gavage was performed from day 5 until day 10 p.i. and the experiment lasted until day 30 p.i. The EAE disease course in imatinib-treated rats was significantly milder from day 9 until day 17 p.i. It seems that the termination of the imatinib treatment on day 10 p.i. resulted in a progression of the disease and one week later the disease severity remained comparable in both groups until the end of the experiment (Fig. 6 A). In the second experimental setup, imatinib was administered continuously from day 5 p.i. until the end of the experiment (Fig. 6 B). Imatinib-treated rats exhibited significantly milder disease symptoms from day 10 p.i. until the end of the experiment. As in the first EAE experimental setup, the onset of the disease was delayed in the imatinib-treated group. Additionally, both cumulative and maximum EAE scores were reduced in the imatinib-treated group, compared to the PBS group (Fig. 6 C). Histopathological analysis performed on the brain and the spinal cord material at day 10 p.i. and 14 p.i., revealed that imatinib-treated rats developed milder inflammation, demyelination and recruited lower amounts of inflammatory cells to the CNS. Both inflammatory index (I.I.) and demyelination score (DM) were significantly lower in imatinib-treated rats compared to the controls, in both time-points analyzed (Fig. 6 E, F), which support our interpretation of reduced chemoattractant expression leading to less recruitment of inflammatory cells to the CNS. Taken together, the imatinib group exhibited generally milder neuroinflammation as well as a delay of the disease onset and the clinical score corresponded to a lower size and reduced number of demyelinated CNS lesions.

Finally, to investigate a possibility for using imatinib as a therapy in the relapsing-remitting phase of MS, gavage began one day after the clinical disease onset (EAE score 1). Indeed, the rats treated with imatinib showed significantly milder disease severity than controls receiving PBS (Fig. 6 D). This indicates that imatinib is a potent therapeutic agent against neuroinflammation, as it ameliorates EAE even after the disease onset. Our findings show that imatinib protects against the MS-like experimentally induced neuroinflammation by improving the BBB integrity, shifting the peripheral immune response towards an anti-inflammatory phenotype, and by interfering with leucocyte chemotaxis.

## Discussion

BBB disruption occurs in diverse CNS disorders such as stroke, Alzheimer’s disease or MS [3], [9], [20], [21]. Here we demonstrate that the small tyrosine kinase inhibitor imatinib ameliorates MS-like neuroinflammation by acting both on the peripheral immune response and the maintenance of the BBB. Imatinib treatment increased the BBB integrity, which was accompanied by lower immune cell infiltration in the CNS. *In vivo* analysis with fluorescently labeled tracer demonstrated that the BBB was tighter in imatinib-treated rats than in the controls. This was additionally supported by less abundant dysferlin+ leaky blood vessels in the imatinib-treated group. Furthermore, occludin, a structural component of BBB endothelial cells was better preserved in imatinib treated rats, indicating increased preservation of endothelial tight junctions.

In this study we provide evidence that imatinib protects against neuroinflammation in the MOG-induced EAE, an animal model of MS. Imatinib treatment suppressed the peripheral immune response, reflected in a shift towards an anti-inflammatory phenotype and an altered cytokine production. Expression array analysis revealed that chemotaxis of immune cells was generally downregulated in the imatinib-treated rats. Specifically, leucocytes showed decreased migration, recruitment and influx to the CNS. We also detected a broad downregulation of chemokines and their receptors which play a role in the attraction of T-cells, such as CXCR1, CX3CR1 and CXCR3 [22]. Concurrently, increased transcript levels of CCL11 were detected, a chemokine known to be important for Th2 recruitment and signaling and protection against EAE [18], [19]. Differential cytokine expression may lead to different activation pattern, for example STAT6/GATA3 and T-bet/STAT1 support Th2 and Th1 differentiation, respectively. Our qPCR data confirmed that *IL4* and *Stat6*, both key regulators of the anti-inflammatory Th2 response, were strongly upregulated in imatinib-treated rats. MOG re-stimulation assay performed with *ex vivo* spleenocytes furthermore strengthened our finding that imatinib supports Th2-like T-cell response. Thereby, IFNγ, TNFα and IL2, all Th1 specific cytokines were downregulated, whereas IL10 and IL5, both Th2 specific cytokines, showed a trend to be upregulated in the imatinib group. It would be interesting to elucidate whether the MAPK or PI3K signalling pathways are modulated upon binding of imatinib to PDGFR-α. A crosstalk via Grb2/Sos and subsequently Ras signalling could modulate the expression of different interleukins such as IL2. However, further studies are required to elucidate how imatinib exactly modulates T-cell differentiation.

The observed shift in the cytokine/chemokine profile resulted in a decreased proliferation of IFNγ-producing MOG-specific T-cells in imatinib-treated rats. Thus, an already dampened immune response in the periphery resulted in a decreased recruitment of T-cells to the CNS, verified by IHC analyses. Interestingly, we observed a subset of imatinib-treated animals harboring a regular amount of macrophages in their inflammatory lesions, while the number of recruited T-cells was surprisingly low. Thus, in experimentally induced MS-like neuroinflammation (EAE), imatinib modulated the T-cell response resulting in less CNS infiltration. Imatinib ameliorated the pathogenic T-cell response, skewed it towards an anti-inflammatory phenotype and shifted the chemokine pattern by downregulating molecules involved in attraction of migrating T-cells. Furthermore, imatinib influenced the communication between the innate and the adaptive immune system, which was reflected by a broad downregulation of many Toll-like receptors (Tlrs). In addition, *IL17* implicated in the initiation phase of EAE, as well as *IL12b* and *IL18*, mediators between the innate and adaptive immune system [23], [24], were downregulated in response to imatinib treatment. Another important mechanism by which imatinib ameliorated the disease was by its action on antigen presenting cells (APC). This was supported by the observation that CSF1R, a key molecule for macrophage function and differentiation was significantly downregulated in the draining lymph nodes, as well as the above mentioned chemokine receptors CXCR1 and CX3CR1. Moreover, IF analysis performed in the spinal cord tissue confirmed the significantly lower amount of MHC class II expression in the imatinib group.

Microarray analysis performed on the peripheral lymph nodes revealed a 4-fold downregulation of *CCR2* in the imatinib-treated rats. Moreover, low expression of the CCR2 protein in the CNS of imatinib-treated rats was confirmed by IHC analysis. CCR2−/− mice are resistant to EAE [16], [17]. It is known that a number of different cell types express CCR2 including monocytes, dendritic cells, basophils, different subset of T-cells, especially Th17 cells, as well as brain resident cells including neurons, astrocytes, brain endothelial cells and microglia [25], [26]. Recently it was shown that CCR2+/CD11b+/Ly-6Chi expression is important for EAE pathogenesis [27]. However, an earlier study showed exactly the opposite, namely an oppressive effect of these monocyte cell subtype [28]. Thus, it would be tempting to elucidate the exact mechanism by which imatinib affects CCR2 expression and which CCR2 carriers are particularly targeted. One potential cell candidate could indeed be CCR2+/CD11b+/Ly-6Chi, as CCR2 is essentially important for the recruitment of monocytes into CNS during EAE [27]. This assumption was also supported by downregulation of CSF1R upon imatinib treatment we observed in the lymph nodes on day 7 p.i. Thus, besides decreasing cell proliferation and activation via downregulating CSF1R, imatinib could also affect migratory properties of monocytes/macrophages by downregulating their CCR2 expression. Together, these effects could possibly account for generally lower disease incidence and/or severity observed in the imatinib-treated group. To this end, it has been recently shown that sorafenib and GW2580, both small molecule tyrosine kinase inhibitors, besides being able to ameliorate EAE in mice even more effective than imatinib, they could suppress M-CSF or PDGF-BB induced TNFα production by macrophages *in vitro* [29]. Besides macrophages, Th17 cells could also be potentially targeted by imatinib, as these cells also express CCR2 [30] and have an important role in EAE pathogenesis [31].

Analyses of EVF isolated from the mice spinal cords day 13 p.i. demonstrated that imatinib did not affect the upregulation of ICAM-1 and VCAM-1 at the BBB, as the expression levels were comparable in both experimental and control group. However, imatinib managed to remarkably normalize the expression of P-selectin, CCL2, CCL19 and CXCL2. These data strongly suggest that imatinib inhibits T-cell entry into the CNS by downregulating expression of adhesion molecules directly at the BBB, in addition to modulating T-cell activation in the peripheral lymphoid organs. Thus, according to our data, imatinib targets both peripheral immune system and the BBB. However, the effect on the BBB level might be secondary to the one observed on the peripheral immune system. The altered expression of adhesion molecules on endothelial cells strongly suggest that imatinib targets PDGFR-α signaling directly at the BBB level, but it could also be the result of a less activated peripheral immune system. T-cell transfer from imatinib-treated animals into untreated recipients and vice versa would probably be the most reliable way to elucidate to what extent the action of imatinib on the peripheral immune system contributes to the effect observed on the BBB. However, according to our data, T-cells from imatinib-treated donors would presumably maintain their anti-inflammatory phenotype upon transfer into recipients for approximately one week (disease protective effect remained significant for one week after termination of the imatinib treatment in Fig. 6 A).

It has been shown that imatinib targets PDGFR-α expressed on astrocytic end-feet in the BBB, counteracting BBB breakdown under pathological conditions [9]. By specifically blocking PDGFR-α signaling, via blocking antibodies against its ligand PDGF-CC, or with imatinib, we previously showed that experimentally induced stroke could be ameliorated [9]. This effect was mediated by a reduction of BBB permeability, which resulted in decreased stroke volume and less hemorrhagic complications. In addition, we have shown that imatinib improves the functional outcome after spinal cord injury by reducing vascular leakage. Two months post-injury, there was a significant preservation of tissue and an enhanced motor function recovery in imatinib-treated rats [32]. Besides PDGFR-α, imatinib can also inhibit PDGFR-β and a recently published study showed that increased PDGFR-β signaling can trigger a diverse subset of immune response genes and thereby increase leucocyte trafficking into the CNS [33]. Thus, future studies should elucidate to what extent PDGFR-α or -β signalling are involved in the process of neuroinflammation and whether it is necessary to target both receptors for successful treatment.

Recently, a randomized pilot study in humans showed that masatinib, a PDGFR, c-kit and Fgf3 inhibitor, has a moderate effect on primary progressive MS (PPMS) and relapse-free secondary progressive MS (rfSPMS) [34]. However, the effect on MS was not statistically significant, due to a huge variation of masatinib effect on the multiple sclerosis functional composite score (MSFC). Therefore, we believe that the clinical trial with imatinib in PPMS and rfSPMS patients would be more successful. It is known that mast cells play an important role in the EAE pathogenesis and masatinib is a potential inhibitor of the mast cell signalling [35], [36]. As we did not observe any particular effect of imatinib on mast cells, one could speculate that a potential combination of imatinib and masatinib in treating neuroinflammation might have a higher therapeutic effect than the monotherapy.

Together with our previously published studies showing that imatinib can successfully block PDGFR-α on the BBB and thereby significantly reduce the stroke volume [9], we now provide evidence that imatinib is also a potent agent when used as a therapy against CNS inflammatory diseases such as MS. Beside affecting the property of the BBB, we show that imatinib potentially influences the peripheral response to specific antigen by skewing T-cell response towards Th2 phenotype. However, imatinib did not seem to affect the homing of the naïve T-cells to the local draining lymph nodes.

Neuroinflammation is a self-sustaining process with the BBB acting as a central regulator and here we show that imatinib can be used for modulating the mechanisms that control the function and integrity of the BBB under pathological conditions. Therefore, we assume that imatinib could potentially be used not only for treating MS, but also for other CNS diseases with an inflammatory component.

## Supporting Information

Figure S1 **No effect of imatinib on mast cell density.** Inguinal lymph nodes and spleens were harvested day on 7 p.i and stained with toluidine blue to visualize mast cells (A). No significant difference in mast cell density in both lymph nodes and spleens from imatinib or PBS treated mice (B–C). Imatinib or PBS was administered via oral gavage from day 2 p.i. until the end of the experiment. Scale bar, 50 µm. n = 8 mice in each group. Error bars represent S.E.M., statistics were calculated using the t-test and *P* values <0.05 were considered significant (*P*<0.05 = *, *P*<0.005 = **, *P*<0.0005 = ***).
(TIF)
