# Correction: Imatinib Ameliorates Neuroinflammation in a Rat Model of Multiple Sclerosis by Enhancing Blood-Brain Barrier Integrity and by Modulating the Peripheral Immune Response

**DOI:** 10.1371/annotation/dd60f248-ca3f-4824-a3b4-c07aefb1c7c3

**Published:** 2013-06-27

**Authors:** Milena Z. Adzemovic, Manuel Zeitelhofer, Ulf Eriksson, Tomas Olsson, Ingrid Nilsson

The name of the first author was listed incorrectly. The correct name is: Adzemovic MV. 

The correct citation is: Adzemovic MZ, Zeitelhofer M, Eriksson U, Olsson T, Nilsson I (2013) Imatinib Ameliorates Neuroinflammation in a Rat Model of Multiple Sclerosis by Enhancing Blood-Brain Barrier Integrity and by Modulating the Peripheral Immune Response. PLoS ONE 8(2): e56586. doi:10.1371/journal.pone.0056586. 

The correct abbreviation in Contributions is: MZA. 

