# Correction: Imatinib Ameliorates Neuroinflammation in a Rat Model of Multiple Sclerosis by Enhancing Blood-Brain Barrier Integrity and by Modulating the Peripheral Immune Response

**DOI:** 10.1371/journal.pone.0123142

**Published:** 2015-04-07

**Authors:** The PLOS ONE Staff

There is an error in the author name in the correction published on June 27, 2013. The correct name is Adzemovic MZ. The correct citation is: Adzemovic MZ, Zeitelhofer M, Eriksson U, Olsson T, Nilsson I (2013) Imatinib Ameliorates Neuroinflammation in a Rat Model of Multiple Sclerosis by Enhancing Blood-Brain Barrier Integrity and by Modulating the Peripheral Immune Response. PLoS ONE 8(2): e56586. doi:10.1371/journal.pone.0056586. The publisher apologizes for the error.
