# Supplementary figures and images for: Imatinib Ameliorates Neuroinflammation in a Rat Model of Multiple Sclerosis by Enhancing Blood-Brain Barrier Integrity and by Modulating the Peripheral Immune Response
Source: PLoS One. 2013 Feb 20;8(2):e56586. doi: 10.1371/journal.pone.0056586 (PMC3577871; doi:10.1371/journal.pone.0056586)

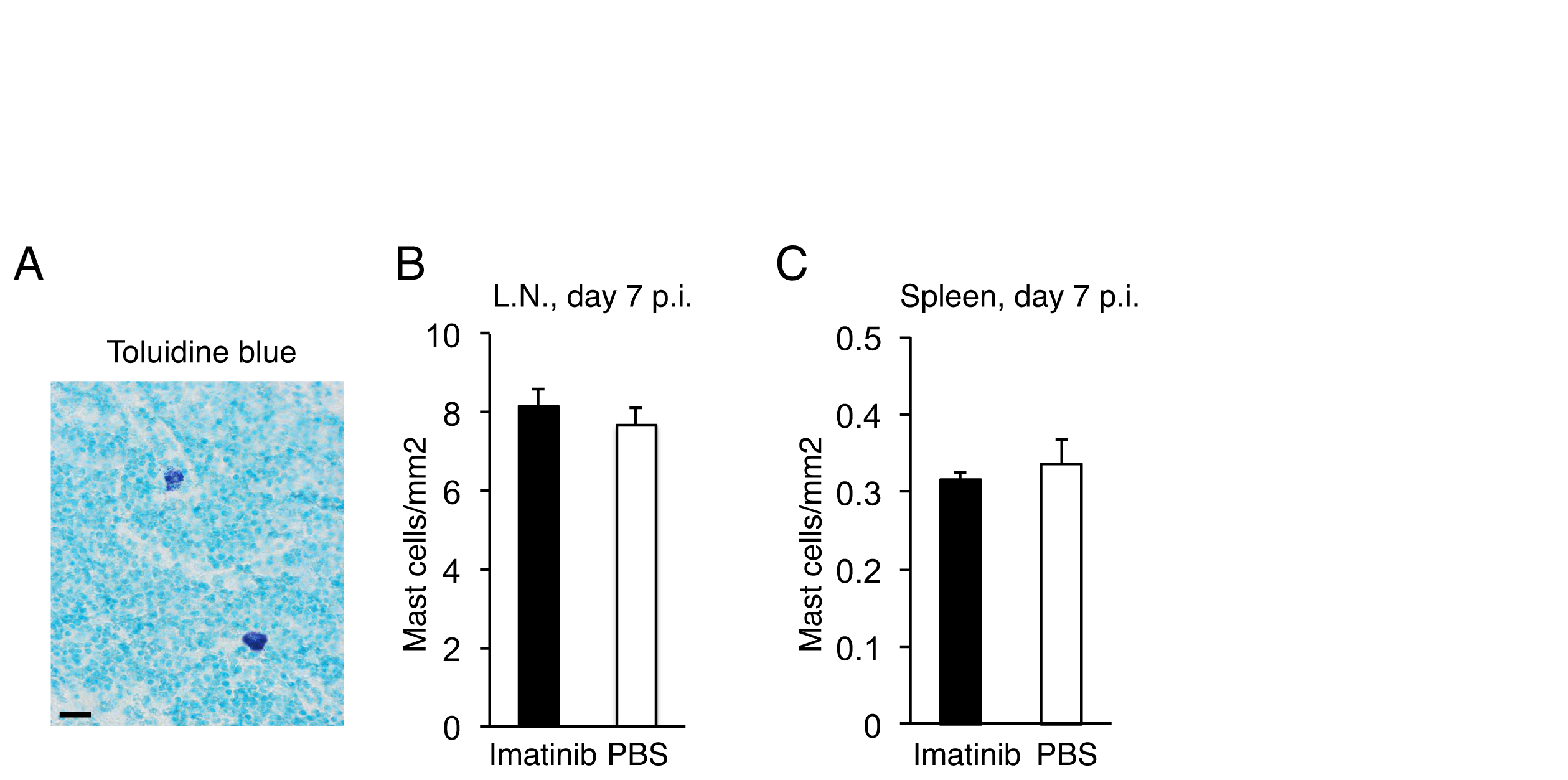

Supplement: Figure S1 — No effect of imatinib on mast cell density. Inguinal lymph nodes and spleens were harvested day on 7 p.i and stained with toluidine blue to visualize mast cells (A). No significant difference in mast cell density in both lymph nodes and spleens from imatinib or PBS treated mice (B–C). Imatinib or PBS was administered via oral gavage from day 2 p.i. until the end of the experiment. Scale bar, 50 µm. n = 8 mice in each group. Error bars represent S.E.M., statistics were calculated using the t-test and P values <0.05 were considered significant (P<0.05 = *, P<0.005 = **, P<0.0005 = ***). (TIF) [file pone.0056586.s001.tif]
